# Enhanced Luminescence and Thermal Stability in High Gd^3+^/Eu^3+^ Co-Doped Ba_3_Y_4_O_9_ Phosphors via Co-Precipitation Method

**DOI:** 10.3390/molecules30051085

**Published:** 2025-02-27

**Authors:** Dong Zhu, Chunfeng Wang, Xiaohuai Wang, Shun Han, Yuxiang Zeng, Ming Fang, Wenjun Liu, Deliang Zhu, Peijiang Cao, Youming Lu

**Affiliations:** 1 College of Materials Science and Engineering, Guangdong Research Center for Interfacial Engineering of Functional Materials, Shenzhen University, Shenzhen 518060, Chinaymlu@szu.edu.cn (Y.L.); 2 College of Physics and Optoelectronic Engineering, Shenzhen University, Shenzhen 518060, China; 3 Department of Physics and Electronic Engineering, Hanshan Normal University, Chaozhou 521041, China

**Keywords:** Gd^3+^ doping, thermal stability, luminescence enhancement, energy transfer

## Abstract

The co-precipitation method was successfully used to synthesize Ba_3_(Y_0.6−x_Gd_0.4_Eu_x_)_4_O_9_ (0.01 ≤ x ≤ 0.09) phosphors with heavy Gd^3+^ doping, resulting in significantly enhanced thermal stability and luminescence performance. Structural analyses confirm that Gd^3+^ and Eu^3+^ ions substitute Y^3+^ in the lattice, causing lattice expansion and improving crystal asymmetry, which enhances Eu^3+^ emission. The incorporation of Gd^3+^ creates efficient energy transfer pathways to Eu^3+^ while suppressing non-radiative relaxation, leading to stable fluorescence lifetimes even at elevated temperatures. With a thermal activation energy of ~0.3051 eV, the Ba_3_(Y_0.55_Gd_0.4_Eu_0.05_)_4_O_9_ phosphor exhibits superior resistance to thermal quenching compared to Ba_3_(Y_0.95_Eu_0.05_)_4_O_9_ and many conventional red phosphors. Furthermore, the reduced color temperature and stable emission spectra across a wide temperature range highlight its potential for advanced lighting and display technologies in high-temperature environments.

## 1. Introduction

Inorganic phosphors play a crucial role in white-light-emitting diodes (W-LEDs) due to their high luminous efficiency, strong brightness, and long operational lifespan, making them indispensable in the lighting industry. The most advanced commercial W-LEDs typically combine gallium nitride (GaN) blue LED chips with Y_3_Al_5_O_12_: Ce^3+^ yellow phosphors [1,2,3]. However, these systems face limitations, including a low color rendering index and high correlated color temperature (CCT), primarily due to insufficient red emission from the phosphor and the dominance of blue light emitted by the LED chip [4,5]. Moreover, the performance of red phosphors often deteriorates under high-temperature conditions, as their emission intensity is affected by radiation transitions and the thermal effects of the chip, further limiting their practical applications in scenarios requiring vivid color rendering. To overcome these challenges, recent research has focused on UV-excited phosphors, utilizing red, green, and blue tricolor emissions to generate white light [6,7,8]. By optimizing doping levels and selecting appropriate tricolor phosphor combinations, W-LEDs with low CCT, high brightness, and superior color quality can be achieved [9,10,11,12]. Enhancing the red emission intensity and thermal stability of phosphors remains a critical objective for advancing next-generation W-LED technologies suitable for high-performance lighting applications.

Phosphor materials for W-LEDs generally feature an inert host lattice that provides a stable luminescent environment, with optically active ions serving as activators. Ba_3_Y_4_O_9_ (BYO) has emerged as a promising host material for red phosphors due to its unique physicochemical properties [13,14,15,16]. Compared to conventional hosts like Y_2_O_3_ and Gd_2_O_3_, BYO offers superior structural stability, an optimal bandgap (~3.436 eV), lower phonon energy, and a higher proportion of asymmetric lattice sites, making it highly compatible with Eu^3+^ doping [17,18]. The 4f energy levels of Eu^3+^ lie outside BYO’s band structure and align well with its electronic configuration, facilitating the formation of discrete luminescent centers [19]. However, the luminescence efficiency of phosphors decreases significantly at elevated temperatures, limiting its practical application in high-temperature environments [20]. Enhancing the thermal stability and high-temperature performance of BYO:Eu^3+^ is therefore essential for advancing its utility in lighting technologies.

The absorption spectrum of Eu^3+^ primarily involves 4f-4f transitions. These transitions are parity-forbidden for electric dipole transitions and only allow weak magnetic dipole transitions, resulting in narrow bands and low absorption efficiency [21,22,23]. When the electrons in the 2p orbitals of ligand oxygen (O^2−^) are transferred to the partially filled 4f orbitals of Eu^3+^, a broad charge transfer band (CTB) appears in the excitation spectrum, significantly enhancing the absorption of excitation energy [24,25]. However, due to the limited intensity of f-f transitions in rare-earth ions, the direct excitation efficiency of Eu^3+^ is low. To address this, Gd^3+^ is introduced as a sensitizer, whose characteristic excitation peaks overlap with the CTB of Eu^3+^, enabling efficient energy absorption and transfer, thereby enhancing the luminescence efficiency of Eu^3+^ [26]. With its half-filled f^7^ configuration, Gd^3+^ exhibits ^8^S_7_/_2_→^6^I_j_ and ^8^S_7_/_2_→^6^P_j_ transitions that partially overlap with the CTB of Eu^3+^, facilitating efficient energy transfer. Li et al. demonstrated that Eu^3+^ emission behavior in Y_2_O_3_ and (Y_0.75_Gd_0.25_)_2_O_3_ revealed this overlap but lacked a mechanistic model for explanation [27]. Mancic et al. demonstrated that substituting Y^3+^ with Gd^3+^ in LnTeBO_5_ stabilized the lattice, reduced the charge transfer barrier, and improved luminescence intensity and thermal stability, though the thermal activation energy remained low at ~0.26 eV [28]. In this study, BYGO:Eu^3+^ (Ba_3_(Y_0.6−x_Gd_0.4_Eu_x_)_4_O_9_, 0.01 ≤ x ≤ 0.09) precursor materials were synthesized via a co-precipitation method, and rapid high-temperature annealing successfully stabilized a high Gd^3+^ doping concentration of 40 a.t.% within the BYO lattice, far exceeding the reported limit of 12% [29]. By analyzing the characteristic emission peaks of Gd^3+^ and Eu^3+^, an energy transfer model from Gd^3+^→Eu^3+^ was established. Thermal quenching experiments demonstrated excellent thermal stability, while the consistent CCT values at elevated temperatures further confirmed that BYGO:Eu^3+^ phosphors are promising candidates for W-LED applications in high-temperature environments.

## 2. Results and Discussion

**Figure 1:**
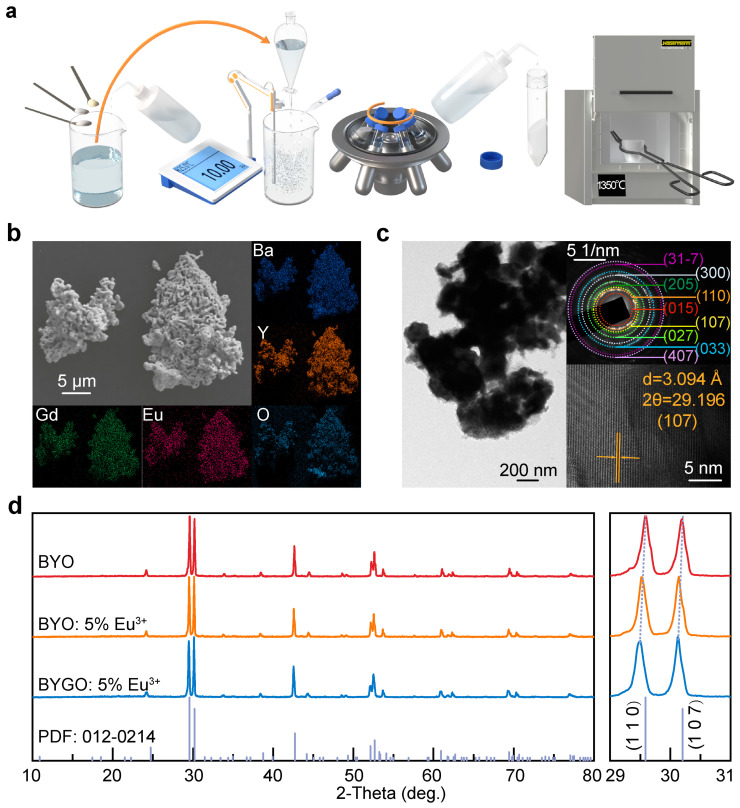
Synthesis and characterization of BYGO:Eu^3+^. (**a**) Synthesis process of BYGO:Eu^3+^ phosphor. (**b**) FE-SEM and EDS mapping of BYGO:Eu^3+^ phosphor. (**c**) FE-TEM analysis of BYGO:Eu^3+^ phosphor. (**d**) XRD pattern of BYGO:Eu^3+^ phosphor, with an enlarged detail of the main peak on the right.

Figure 1a illustrates the synthesis process of BYGO:Eu^3+^ phosphors using a co-precipitation method. First, precursor solutions were prepared in specific proportions and fully dissolved. These solutions were then slowly added dropwise into an ammonium bicarbonate solution using a separatory funnel. Throughout the titration process, the solution’s pH was continuously monitored with a pH meter and maintained at 10 by adding ammonium hydroxide. After titration, the mixture was stirred for an additional 6 h to ensure a complete reaction. The resulting precipitate was washed multiple times, with the final wash performed using n-hexane to remove any residual organic impurities. The washed product was dried at 60 °C for 12 h in an oven to obtain the precursor. This precursor was then calcined at a ramping rate of 1 °C/min to 1350 °C, held at the target temperature for 6 h, and rapidly cooled to room temperature to yield the BYGO:Eu^3+^ phosphors. Figure 1b shows the SEM images of the BYGO:Eu^3+^ phosphors synthesized by the co-precipitation method. The phosphor particles are approximately 1 μm in size, exhibiting irregular shapes and noticeable voids between particles. These voids are attributed to the decomposition of HCO_3_^−^ and CO_3_^2−^ in the precursor during heating [30]. The connections between particles result from the sintering process, where ~40 nm precursor particles underwent grain growth during high-temperature calcination (Figure S1). Energy-dispersive spectroscopy (EDS) mapping confirms the uniform distribution of Ba, Y, Gd, Eu, and O elements throughout the phosphors, which facilitates more efficient luminescence performance.

Figure 1c presents high-resolution transmission electron microscopy (HR-TEM) images of BYGO:Eu^3+^ phosphors. The particles exhibit slight aggregation, and their size aligns with the SEM results. The selected area electron diffraction (SAED) pattern reveals bright spot rings, indicating high crystallinity and confirming the formation of a polycrystalline BYO host structure consistent with JCPDS No.012-0214. In the HRTEM images of the optimized nanoscale samples, distinctive lattice fringes are observed with an interplanar spacing of 3.094 Å, closely matching the standard value of 2.9754 Å for the (1 0 7) crystal plane of the Ba_3_Y_4_O_9_ host. However, the spacing is increased by ~0.1186 Å, indicating a lattice expansion of approximately 4.0%. This expansion results from the substitution of Y^3+^ ions with larger Gd^3+^ and Eu^3+^ ions in the BYO lattice. Figure 1d displays the XRD patterns of undoped and doped BYO samples. All patterns match well with the standard Ba_3_Y_4_O_9_ data, with no additional diffraction peaks observed, confirming that the dopant ions were fully incorporated into the BYO lattice without forming impurity phases. The magnified main peak detail on the right further reveals that, with the doping of Eu^3+^ and Gd^3+^, all diffraction peaks shift toward smaller angles, accompanied by a deterioration in crystallinity. This indicates that the excessive substitution of the original lattice Y^3+^ ions by the larger Gd^3+^ and Eu^3+^ ions leads to lattice expansion and structural changes.

**Figure 2:**
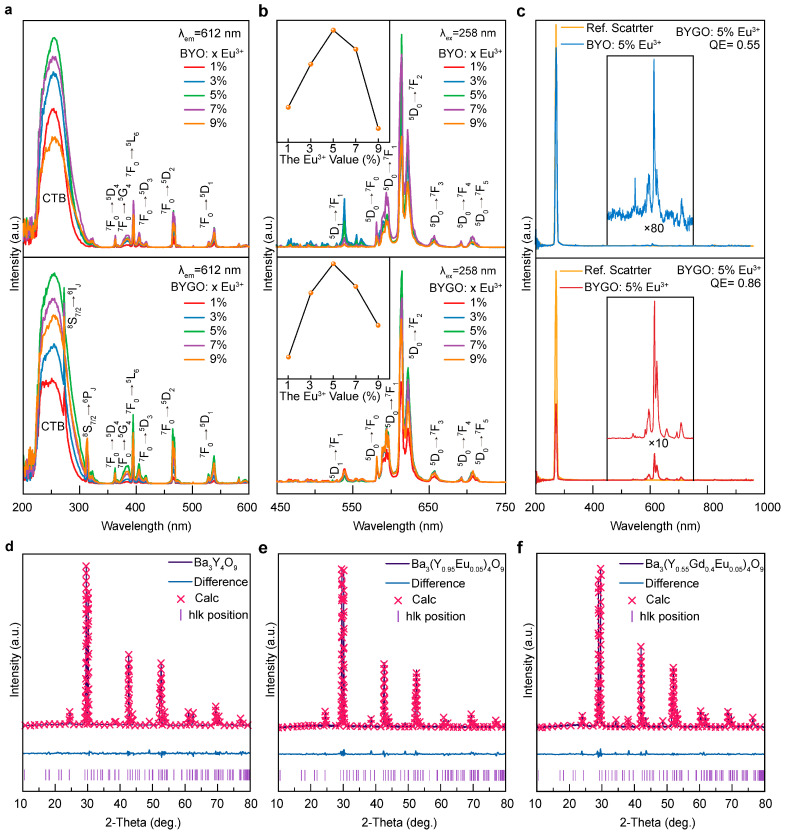
Optical characterization of BYGO:Eu^3+^ phosphor. (**a**) PLE spectra of phosphors BYO: x% Eu^3+^ and BYGO: x% Eu^3+^ (x = 1, 3, 5, 7, 9). (**b**) PL spectra of phosphors BYO: x% Eu^3+^ and BYGO: x% Eu^3+^ (x = 1, 3, 5, 7, 9). The inset shows the variation of PL intensity with Eu^3+^ concentration under 258 nm excitation. (**c**) Comparison of quantum efficiency between BYO: 5% Eu^3+^ and BYGO: 5% Eu^3+^, with the inset showing the magnified details of their respective quantum efficiency. (**d**) Rietveld refinement profile of BYO. (**e**) Rietveld refinement profile of BYO: 5%Eu^3+^. (**f**) Rietveld refinement profile of BYGO: 5%Eu^3+^.

Figure 2a presents the PLE spectra of Eu^3+^-doped BYO and BYGO systems, showing two primary components. The first component is a broadband excitation peak centered at 258 nm (CTB), spanning the 220–330 nm short-wavelength region. This peak arises from the charge transfer transition of electrons from the 2p orbitals of O^2−^ to the empty 4f orbitals of Eu^3+^, forming an excited state [24]. The second component consists of several sharp excitation peaks in the 313–538 nm long-wavelength region, corresponding to the 4f-4f transitions of Eu^3+^. These include ~363 nm (^7^F_0_→^5^D_4_), ~385 nm (^7^F_0_→^5^G_4_), ~395 nm (^7^F_0_→^5^L_6_), ~417 nm (^7^F_0_→^5^D_3_), ~465 nm (^7^F_0_→^5^D_2_), and ~538 nm (^7^F_0_→^5^D_1_) [31]. Among these, the CTB intensity is significantly higher than that of the intra-4f transitions, indicating that CTB excitation is the most effective method to achieve fluorescence in the Eu^3+^-doped BYO system. Notably, the peak at 394 nm (^7^F_0_→^5^L_6_) exhibits the highest intensity among the 4f-4f transitions [32]. This hypersensitive transition is highly dependent on the strength of the crystal field, meaning even minor variations in the local structure or surrounding environment of Eu^3+^ can significantly affect its intensity. Additionally, two extra peaks at 275 nm and 315 nm appear in the BYGO system, attributed to the ^8^S_7/2_→^6^I_J_ and ^8^S_7/2_→^6^P_J_ transitions of Gd^3+^. The 275 nm peak overlaps with the CTB, enabling efficient energy transfer from Gd^3+^→Eu^3+^ [33]. Figure 2b shows the PL spectra of Eu^3+^-doped BYO and BYGO systems under 258 nm (CTB) excitation. Six emission peaks are detected, with the most prominent one at ~612 nm (^5^D_0_→^7^F_2_), which corresponds to an electric dipole transition [34]. This transition is highly sensitive to lattice asymmetry due to the symmetry-breaking effect of the 4f orbitals in non-centrosymmetric environments. The split peaks in the orange region (~589 nm, ~595 nm, and ~601 nm) result from the magnetic dipole transition (^5^D_0_→^7^F_1_), which occurs in centrosymmetric environments. The asymmetry of the Eu^3+^ local environment is reflected in the intensity ratio (IR/O) of the ^5^D_0_→^7^F_2_ and ^5^D_0_→^7^F_1_ transitions. In the BYO system, the IR/O value is 2.92, indicating a highly asymmetric local environment for Eu^3+^ ions. In the BYGO system, the IR/O ratio increases slightly (~3.06), attributed to the lattice expansion caused by the larger ionic radius of Gd^3+^ (~1.053 Å, CN = 8) compared to Y^3+^ (~1.040 Å, CN = 8), which enhances asymmetry. Additionally, as a common sensitizer, Gd^3+^ significantly enhances Eu^3+^ emission, increasing the overall PL intensity of BYGO by ~135% compared to BYO under identical conditions (Figure S2). The inset shows the emission intensity trend for different Eu^3+^ doping concentrations, consistent with previously reported studies [35].

Figure 2c evaluates the quantum efficiency (QE), a critical parameter determining phosphor brightness (see Note S1 for QE calculation) [36,37]. For BYO and BYGO systems, the maximum QE is achieved at the quenching concentration (~5% Eu^3+^), reaching ~55% and ~86%, respectively (Figure S3). The QE trends align with those of the PL intensity, confirming that doping concentration strongly influences luminescence properties. In the Gd^3+^/Eu^3+^ co-doped systems, Martins et al. measured a quantum efficiency (QE) of 48% in Y_2_O_3_ [38], while Liu et al. reported a QE of 70.6% in LiGd_0.5_Eu_0.5_MgWO_6_ [39], both of which are lower than that of BYGO system.

To confirm the substitution of Y^3+^ by Gd^3+^ in the BYO lattice, Figure 2d–f presents the Rietveld refinement results for BYO, BYO: 5% Eu^3+^, and BYGO: 5% Eu^3+^, analyzed using Topas 3-C software. Comparative analyses of the structural parameters are summarized in Table S1, with refinement parameters detailed in Tables S2–S4 [40]. The results confirm that the ionic radii of Gd^3+^ (~0.938 Å, CN = 6; ~1.053 Å, CN = 8) and Eu^3+^ (~0.950 Å, CN = 6; ~1.066 Å, CN = 8) are larger than that of Y^3+^, making excessive doping prone to introducing impurity phases. However, the use of chemical co-precipitation and rapid cooling at 1350 °C effectively traps impurity ions within the lattice, reducing their escape probability. Calculations indicate that ~4.9 at.% Eu^3+^ and ~38.15 at.% Gd^3+^ successfully replaced Y^3+^ sites, with the lattice volume expanding by ~3.19%. This conclusion is consistent with the lattice expansion observed in the TEM results (Figure 1c).

**Figure 3:**
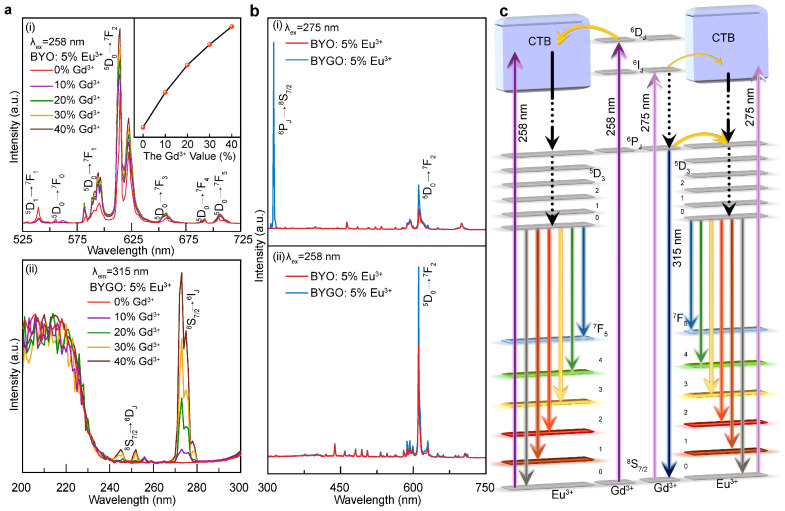
Energy transfer in BYGO:Eu^3+^ phosphor. (**a**) Comparison of PL/PLE spectra: (i) comparison of the PL of BYGO: 5% Eu^3+^ phosphors doped with different Gd^3+^ concentrations, with an excitation wavelength of 258 nm, (ii) comparison of the photoluminescence excitation (PLE) of BYGO: 5% Eu^3+^ phosphors doped with different Gd^3+^ concentrations, with an emission wavelength of 315 nm: (**b**) comparison of the PL spectra of BYO: 5%Eu^3+^ and BYGO: 5% Eu^3+^ at different excitation wavelengths, (i) comparison of PL spectra at an excitation wavelength of 275 nm, (ii) comparison of PL spectra at the normal excitation wavelength of 258 nm. (**c**) Schematic diagram of energy transfer in the BYGO: 5% Eu^3+^ system.

Excessive Gd^3+^ not only affects the lattice sites but also alters the original luminescence process in the BYO:Eu^3+^ system [41]. At high concentrations, Gd^3+^ significantly increases the probability of cross-relaxation between Eu^3+^ ions within the lattice. Li et al. reported that in Eu^3+^/Gd^3+^ co-doped phosphors where Y^3+^ is partially substituted, the emission intensity of Eu^3+^ increases linearly with the Gd^3+^ concentration [42]. This trend is confirmed in Figure 3a(i), where the photoluminescence (PL) intensity of BYGO: 5% Eu^3+^ exhibits a nearly linear enhancement as Gd^3+^ concentration increases. Therefore, the primary objective of this study is to maximize the incorporation of Gd^3+^ into the BYO lattice while avoiding the formation of secondary phases. As shown in Figure S4, when the Gd^3+^ concentration reaches 45%, the structural equilibrium of the crystal is disrupted, leading to the appearance of multiple impurity phases. This is why the Gd^3+^ content in this work is limited to 40%. To investigate the sensitizing role of Gd^3+^ in the BYGO:Eu^3+^ system, we measured its photoluminescence excitation (PLE) spectrum using the characteristic emission wavelength of Gd^3+^ at 315 nm. As depicted in Figure 3a(ii), the characteristic excitation peak of Gd^3+^ (^8^S_7_/_2_→^6^I_j_) appears clearly at 275 nm, while additional excitation peaks corresponding to the ^8^S_7_/_2_→^6^D_j_ transitions are observed at 244 nm and 252 nm. These peaks are absent in BYO:5%Eu^3+^, indicating that they are directly associated with the presence of Gd^3+^. PL spectra under different monitoring wavelengths are presented in Figure 3b. For BYO: 5% Eu^3+^, the PL intensity is weak when the excitation wavelength is at 275 nm, as this wavelength lies within the CTB excitation range of Eu^3+^. In contrast, the PL spectrum of BYGO: 5% Eu^3+^ shows not only the characteristic emission peaks of Eu^3+^ but also a strong emission peak at ~315 nm corresponding to the ^6^P_j_→^8^S_7/2_ transition of Gd^3+^. This indicates that a significant portion of the ultraviolet energy absorbed by the system is released as Gd^3+^ emission, while only a small fraction is transferred to Eu^3+^ through energy transfer, as shown in Figure 3b(i). When the monitoring wavelength is shifted to 258 nm (Figure 3b(ii)), the Gd^3+^ emission peaks are nearly absent, leaving only the strong characteristic emissions of Eu^3+^. This suggests that under 258 nm excitation, nearly all energy in the system is efficiently transferred to Eu^3+^.

Based on the Judd–Ofelt theory, the energy transfer mechanism in the BYGO:Eu^3+^ system is depicted in Figure 3c. At an excitation wavelength of 275 nm, the BYO:Eu^3+^ system shows weak emission because, although this wavelength is not the optimal excitation wavelength, it still falls within the CTB region of Eu^3+^, allowing for low-efficiency photon absorption. In contrast, the BYGO:Eu^3+^ system exhibits a sharp and intense ultraviolet emission (~315 nm). This energy corresponds to the transition of Gd^3+^ from the ^8^S_7/2_→^6^I_J_ energy level, with a portion of the energy transferred to Eu^3+^, as the 6I_j_ level overlaps with Eu^3+^’s CTB. Additionally, some Gd^3+^ ions undergo the ^6^I_J_→ ^6^P_J_ transition, and the energy difference generated in this process is suitable for Eu^3+^’s ^5^D_0_ energy level absorption, promoting the characteristic Eu^3+^ emission [43]. Therefore, as shown in Figure 3b(i), the emission intensity of BYGO:Eu^3+^ at 612 nm is stronger than that of BYO:Eu^3+^. At an excitation wavelength of 258 nm, the BYO:Eu^3+^ system directly absorbs photon energy, with electrons transitioning from the ground state ^7^F_0_ to the CTB and subsequently relaxing non-radiatively to the ^5^D_0_ excited state. This is followed by a radiative transition to the ^7^F_J_ (J = 0, 1, 2, 3, 4) states, emitting orange-red light. In the BYGO:Eu^3+^ system, electrons transition from the ^8^S_7/2_→^6^D_J_, with energy transfer occurring through multiple Gd^3+^ ions in the lattice, concentrating the excitation energy onto a few high-energy Gd^3+^ ions. These high-energy Gd^3+^ ions then efficiently transfer energy to Eu^3+^. The better energy level matching between high-energy Gd^3+^ and Eu^3+^ significantly enhances the energy transfer efficiency from Gd^3+^→Eu^3+^. Therefore, in Figure 3b(ii), the characteristic emission of Gd^3+^ is nearly absent, and only the intense Eu^3+^ emission is observed. These processes work synergistically, achieving efficient energy transfer from Gd^3+^→Eu^3+^ [44].

**Figure 4:**
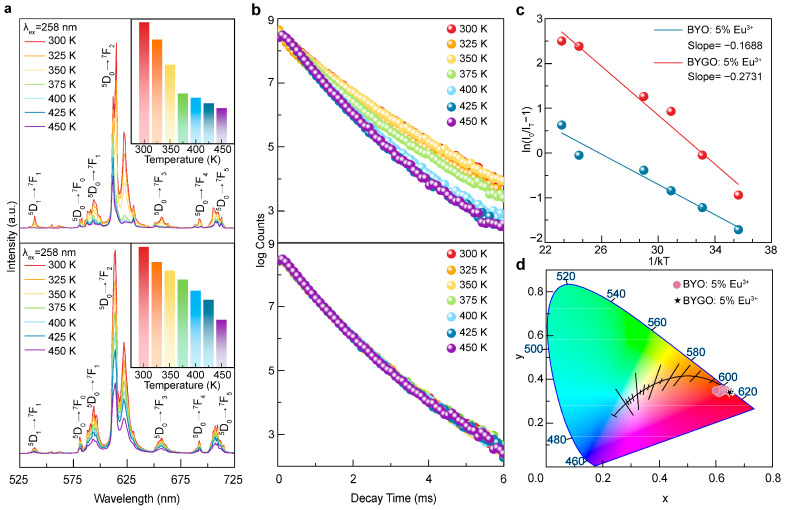
Comparison of luminescent properties of BYO: 5% Eu^3+^ and BYGO: 5% Eu^3+^ at different temperatures. (**a**) PL comparison of BYO: 5% Eu^3+^ and BYGO: 5% Eu^3+^ at different temperatures, with the inset showing the trend of luminescence intensity variation. (**b**) Comparison of decay times for BYO: 5% Eu^3+^ and BYGO: 5% Eu^3+^ at different temperatures. (**c**) Thermal quenching activation energy of BYO: 5% Eu^3+^ and BYGO: 5% Eu^3+^. (**d**) CIE color coordinates of BYO: 5% Eu^3+^ and BYGO: 5% Eu^3+^ at different temperatures.

Phosphor materials are often required to perform under diverse operational environments, with thermal stability being a critical factor, especially at elevated temperatures [45]. Higher temperatures intensify lattice vibrations, increase non-radiative relaxation pathways, and lead to reduced luminescence efficiency and fluorescence lifetime, culminating in thermal quenching [46]. However, the incorporation of substantial amounts of Gd^3+^ has been shown to effectively mitigate these issues. As demonstrated in Figure 4a, the temperature-dependent PL spectra of BYO:Eu^3+^ and BYGO:Eu^3+^ phosphors reveal that at 300 K, the emission intensity of BYO is 53.52% of BYGO. As the temperature increases to 450 K, this ratio decreases to 20.14%, indicating that BYO exhibits a faster decline in emission intensity. Within the practical operating temperature range for LEDs (~400 K), the emission intensity of BYGO retains 59.56% of its initial value at 300 K, whereas BYO retains only 20.26%, clearly highlighting the improved thermal stability conferred by Gd^3+^ doping. To investigate the underlying mechanism, the temperature-dependent fluorescence decay lifetimes of BYO:Eu^3+^ and BYGO:Eu^3+^ phosphors were measured, as shown in Figure 4b. The fluorescence lifetime (*τ*) was calculated using the method outlined in Note S2 [47,48]. As the temperature increases from 300 K to 450 K, under the detection conditions of an excitation wavelength of 258 nm and an emission wavelength of 612 nm, the *τ* of BYO: 5% Eu^3+^ decreases significantly from ~1.038 ms to ~0.774 ms. In contrast, under the same detection conditions, the *τ* of BYGO: 5% Eu^3+^ remains relatively stable at ~0.744 ms. In the undoped BYO:Eu^3+^ system, 2p electrons of O^2−^ transfer energy to Eu^3+^ through the CTB, exciting its 4f states [49]. The energy is subsequently dissipated via phonon-mediated non-radiative relaxation, a process that becomes increasingly pronounced at higher temperatures, leading to shorter excited-state lifetimes and diminished radiative efficiency. In contrast, the introduction of Gd^3+^ induces lattice expansion and increases structural asymmetry, effectively reducing lattice stress and defect density, thereby suppressing multi-phonon relaxation. Furthermore, the efficient energy transfer pathway from Gd^3+^ to Eu^3+^ enhances Eu^3+^ emission intensity while minimizing non-radiative relaxation. This mechanism ensures that the BYGO system exhibits superior fluorescence lifetime stability at elevated temperatures, significantly mitigating thermal quenching effects.

The thermal activation energy (E_a_), calculated using the Arrhenius equation (as shown in Note S3) [49,50], serves as a key parameter for assessing a material’s resistance to thermal quenching. Figure 4c illustrates the E_a_ fitting results for both BYO:Eu^3+^ and BYGO:Eu^3+^ phosphors. Based on these calculations, the E_a_ for BYO:Eu^3+^ is 0.178 eV, whereas for BYGO:Eu^3+^, the E_a_ increases to approximately 0.305 eV. This value surpasses those reported for many red phosphors (see Table S5), underscoring the enhanced thermal stability of the BYGO system due to Gd^3+^ doping [46,50,51,52,53].

To further investigate the effect of Gd^3+^ doping on luminescent properties, Figure 4d illustrates the CIE chromaticity coordinates of BYO: 5% Eu^3+^ and BYGO: 5% Eu^3+^ at different temperatures. As the temperature increases, the CIE coordinates of BYO: 5% Eu^3+^ exhibit significant shifts, moving from the red region at (0.6382, 0.3258) to the orange region at (0.5951, 0.3008). This change is attributed to intensified lattice expansion at elevated temperatures, where the ^5^D_0_→^7^F_2_ electric dipole transition at 612 nm, being more sensitive to the local coordination environment, undergoes faster thermal quenching compared to the ^5^D_0_→^7^F_1_ magnetic dipole transition at ~538 nm. In contrast, BYGO: 5% Eu^3+^ maintains stable CIE coordinates at (0.6524, 0.3471) across the entire temperature range, benefiting from a more stable lattice structure and reduced influence of phonon energy on energy transfer. The CCT values, calculated using the method detailed in Note S4 and summarized in Table S6, further corroborate this stability [37,54,55]. The CCT of BYO: 5% Eu^3+^ varies between 3161 K and 3468 K, while BYGO: 5% Eu^3+^ consistently achieves a lower CCT of approximately 2700 K, which is favorable for improved color rendering [56]. These findings indicate that Gd^3+^ doping not only enhances luminescence intensity and efficiency but also significantly improves lattice stability and thermal resistance. Such advancements provide a novel design approach for lighting applications in dynamic and high-temperature environments.

## 3. Materials and Methods

Barium nitrate (Ba(NO_3_)_2_, 99.999%), yttrium nitrate hexahydrate (Y(NO_3_)_3_·6H_2_O, 99.99%), gadolinium nitrate hexahydrate (Gd(NO_3_)_3_·6H_2_O, 99.99%), and europium nitrate hexahydrate (Eu(NO_3_)_3_·6H_2_O, 99.99%) were procured from Alfa Aesar (China) Chemical Co., Ltd. (Shanghai, China). Ammonium bicarbonate (NH_4_HCO_3_, 99.995%), n-hexane (C_6_H_14_, UV/VIS spectroscopy grade), and absolute ethanol (CH_3_CH_2_OH, 99.8%) were supplied by Shanghai Aladdin Biochemical Technology Co., Ltd. (Shanghai, China). All reagents were of analytical grade and used as received without further purification.

For each synthesis of the BYGO:Eu^3+^ precursor, stoichiometric amounts of nitrate salts were precisely weighed according to their atomic ratios and dissolved in ultrapure water to prepare 250 mL of solution. This solution was slowly added dropwise into an ammonium bicarbonate solution while maintaining a constant pH of 10 by the controlled addition of dilute ammonium hydroxide. After the titration, the suspension was stirred continuously for 6 h to ensure complete homogenization. The resulting precipitate was washed thoroughly with deionized water and n-hexane to remove impurities, followed by drying at 65 °C for 6 h to obtain the precursor powder. The dried precursor was calcined in a muffle furnace (Nabertherm LHT 08-18, Lilienthal, Germany) at a heating rate of 1 °C/min. The temperature was raised to 1350 °C and maintained for 5 h. At the end of the calcination process, the phosphor samples were extracted from the furnace at 1350 °C and rapidly cooled to room temperature to facilitate phase transformation. The cooled phosphor powders were ground finely using an agate mortar and pestle and then sieved through a 1000-mesh stainless steel sieve (15 μm pore size) to achieve uniform particle size distribution.

The surface morphology of the phosphor materials was examined using a field emission scanning electron microscope (FE-SEM, Hitachi SU-70, Tokyo, Japan) equipped with an energy-dispersive spectroscopy (EDS) system operated at an accelerating voltage of 5 kV to analyze elemental composition. The nanoscale characteristics and lattice spacings of the samples were further analyzed by field emission transmission electron microscopy (FE-TEM, JEM-F200, JEOL, Akishima, Japan). Selected area electron diffraction (SAED) patterns were also acquired using the TEM’s integrated detector. X-ray diffraction (XRD) analysis of all samples was performed using a Rigaku SmartLab XRD system(Rigaku Corporation, Bruker D8 Advance, Tokyo, Japan). Scans were conducted from 10° to 90° in 2θ, with a step size of 0.02° and a scanning speed of 0.05 s per step under ambient conditions (Cu Kα radiation, λ = 1.5412 Å). Structural refinement of the XRD data was carried out using the Le Bail method with Topas 3-C software to determine the crystal structure. Photoluminescence (PL) emission, photoluminescence excitation (PLE), and fluorescence decay curves were recorded using an Edinburgh Instruments FLS-1000 fluorimeter (Edinburgh Instruments Ltd., Edinburgh, UK). Quantum efficiency (QE) was measured using a Horiba DeltaFlex instrument (Horiba Scientific, Kyoto, Japan) equipped with a 260 nm NanoLED laser, enabling precise evaluation of the phosphor’s photoluminescent properties.

## 4. Conclusions

A series of BYO:Eu^3+^ phosphors were synthesized via co-precipitation, and doping with ~40% Gd^3+^ led to significant improvements in both luminescent performance and thermal stability. Structural analysis confirmed that Gd^3+^ substitution for Y^3+^ caused a lattice expansion of approximately 4.0%, which enhanced the energy transfer from Gd^3+^ to Eu^3+^, resulting in increased Eu^3+^ emission intensity. The quantum efficiency of BYGO: 5% Eu^3+^ reached ~86%, notably higher than the ~55% observed for BYO: 5% Eu^3+^. Thermal activation energy (E_a_) for BYGO:Eu^3+^ was calculated to be ~0.3051 eV, significantly surpassing the ~0.1688 eV for BYO:Eu^3+^, indicating improved resistance to thermal quenching. Even at elevated temperatures (300 K to 450 K), BYGO:Eu^3+^ maintained a stable fluorescence lifetime (~0.744 ms) and a consistent color temperature (~2066 K), reflecting enhanced high-temperature color stability. These findings demonstrate that Gd^3+^ doping substantially improves the thermal and optical properties of the BYGO:Eu^3+^ system, making it highly promising for high-temperature applications in LED lighting and display technologies.

## Data Availability

The original contributions presented in this study are included in the article/Supplementary Materials. Further inquiries can be directed to the corresponding authors.

## Supplementary Materials

The following supporting information can be downloaded at https://www.mdpi.com/article/10.3390/molecules30051085/s1. Figure S1. FE-SEM images of a single BYGO: 5% Eu^3+^ precursor (a) and the same precursor after calcination at 1350 °C (b), showing significant morphological changes due to thermal treatment; Figure S2. Comparison of emission intensity between Eu^3+^-doped BYO and BYGO systems; Figure S3. Comparison of QE between BYO:Eu^3+^ and BYGO:Eu^3+^; Figure S4. XRD of Ba_3_Y_2_Gd_1.8_Eu_0.2_O_9_; Table S1. Crystal structure data interpretation of Ba_3_Y_4_O_9_, Ba_3_Y_3.8_Eu_0.2_O_9_, and Ba_3_Y_2.2_Gd_1.6_Eu_0.2_O_9_ phosphors with reference to standard Ba_3_Y_4_O_9_ host at room temperature 25 °C; Table S2. Diverse atomic parameters together with the refined atomic positions of Ba_3_Y_4_O_9_ phosphor; Table S3. Diverse atomic parameters together with the refined atomic positions of Ba_3_Y_3.8_Eu_0.2_O_9_ phosphor; Table S4. Diverse atomic parameters together with the refined atomic positions of Ba_3_Y_2.2_Gd_1.6_Eu_0.2_O_9_ phosphor; Table S5. Comparison of activation energy of the BYGO phosphor with some previously reported phosphors; Table S6. Color temperature statistics of the samples; Note S1. Quantum yields; Note S2. Decay time; Note S3. Activation energy; Note S4. Color temperature.

## Abbreviations

The following abbreviations are used in this manuscript:

W-LEDs	White-light-emitting diodes
BYO:Eu^3+^	Ba_3_Y_4−x_O_9_:xEu(0.01 ≤ x ≤ 0.09)
BYGO:Eu^3+^	Ba_3_(Y_0.6_Gd_0.4_)_4_O_9_:xEu(0.01 ≤ x ≤ 0.09)
CCT	Correlated color temperature
CTB	Charge transfer band
PL	Photoluminescence
PLE	Photoluminescence excitation
QE	Quantum efficiency
FE-SEM	Field emission scanning electron microscope
EDS	Energy-dispersive spectroscopy
FE-TEM	Field emission transmission electron microscopy
SAED	Selected area electron diffraction
XRD	X-ray diffraction
PDF	Powder diffraction file
*E_a_*	Activation energy
