# Supplementary material for: Enhanced Luminescence and Thermal Stability in High Gd3+/Eu3+ Co-Doped Ba3Y4O9 Phosphors via Co-Precipitation Method
Source: Molecules. 2025 Feb 27;30(5):1085. doi: 10.3390/molecules30051085 (PMC11901592; doi:10.3390/molecules30051085)
Supplement: Supplementary file 1 [file molecules-30-01085-s001.zip › molecules-3480058-supplementary.pdf]

Supplementary Materials

Enhanced Luminescence and Thermal Stability in High Gd^{3+}/Eu^{3+} Co-Doped $Ba_3Y_4O_9$ Phosphors via Co-Precipitation Method

Dong Zhu [1,2], Chunfeng Wang [1], Xiaohuai Wang [3,*], Shun Han [1], Yuxiang Zeng [1], Ming Fang [1], Wenjun Liu [1], Deliang Zhu [1], Peijiang Cao [1,*] and Youming Lu [1,2,3,*]

[1] *Shenzhen Key Laboratory of Special Functional Materials, Guangdong Research Center for Interfacial Engineering of Functional Materials, College of Materials Science and Engineering, Shenzhen University, Shenzhen 518060, China.*

[2] *Key Laboratory of Optoelectronic Devices and Systems of Ministry of Education and Guangdong Province, College of Physics and Optoelectronic Engineering Shenzhen University, Shenzhen 518060, China.*

[3] *Department of Physics and Electronic Engineering, Hanshan Normal University, Chaozhou 521041, China*

***E-mail:** wxh1997@hstc.edu.cn (Xiaohuai Wang), pjcao@szu.edu.cn (Peijiang Cao) and ymlu@szu.edu.cn (Youming Lu)

Figure S1

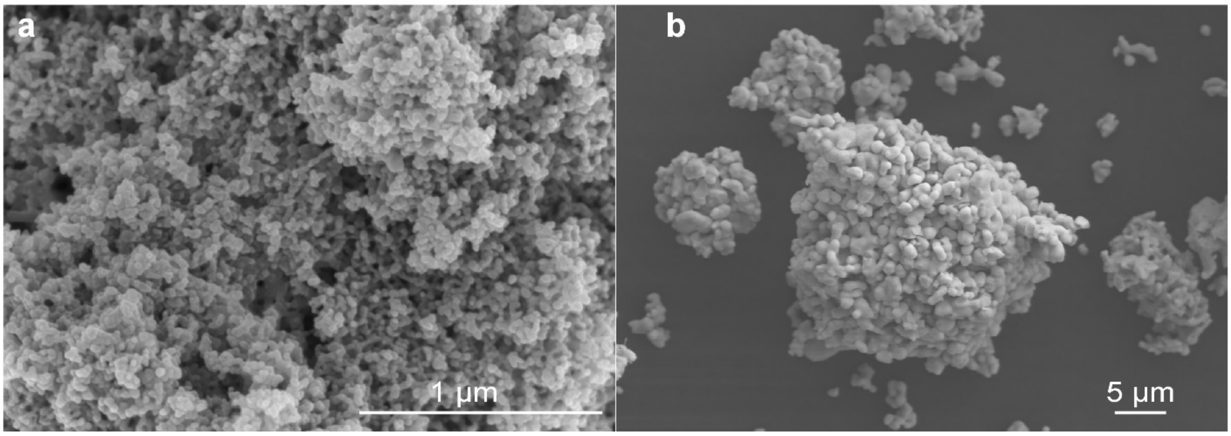

Figure S1 FE-SEM images of a single BYGO: 5% Eu^{3+} precursor (a) and the same precursor after calcination at 1350°C (b), showing significant morphological changes due to thermal treatment.

Figure S1 shows the field-emission scanning electron microscopy (FE-SEM) images of the BYGO: 5% Eu^{3+} precursor (a) after calcination at 1350°C. The precursor particles are very small, with a size of approximately ~40 nm, and exhibit an irregular granular morphology. The product obtained after calcination at 1350°C retains the morphology of the precursor, but the particles have significantly grown in size, and the overall contours have become more distinct.

Figure S2

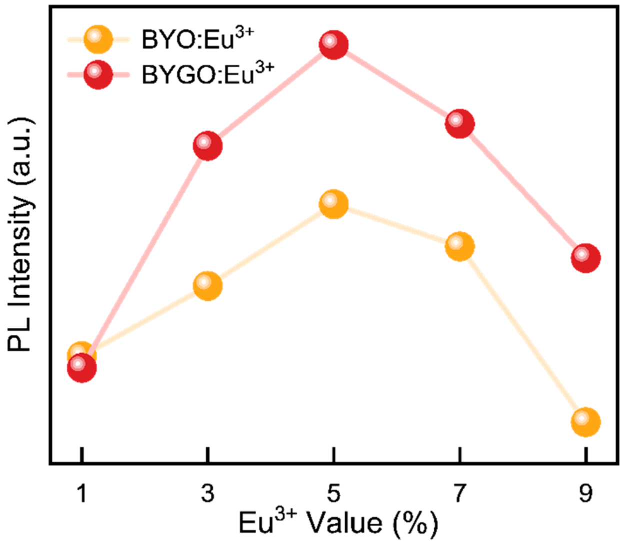

Figure S2 Comparison of emission intensity between Eu^{3+}-doped BYO and BYGO systems.

Figure S2 shows the comparison of the integrated emission intensity values for BYO and BYGO systems doped with different concentrations of Eu^{3+}. The quenching concentration of Eu^{3+} is 5% for both systems. The overall trend indicates that the emission intensity increases with Eu^{3+} concentration until reaching the quenching concentration, after which it begins to decrease. At a low Eu^{3+} concentration of 1%, the emission intensities of both BYO and BYGO systems are nearly identical, as the activator ion concentration is minimal. However, as the activator ion concentration increases, the energy transfer effect from $Gd^{3+} \rightarrow Eu^{3+}$ in the BYGO system becomes significant. Specifically, the emission intensity of BYGO:5%Eu^{3+} is approximately 135% higher than that of BYO:5%Eu^{3+}. In contrast, the emission intensity of BYO:5%Eu^{3+} is even weaker than that of BYGO:3%Eu^{3+}.

Figure S3

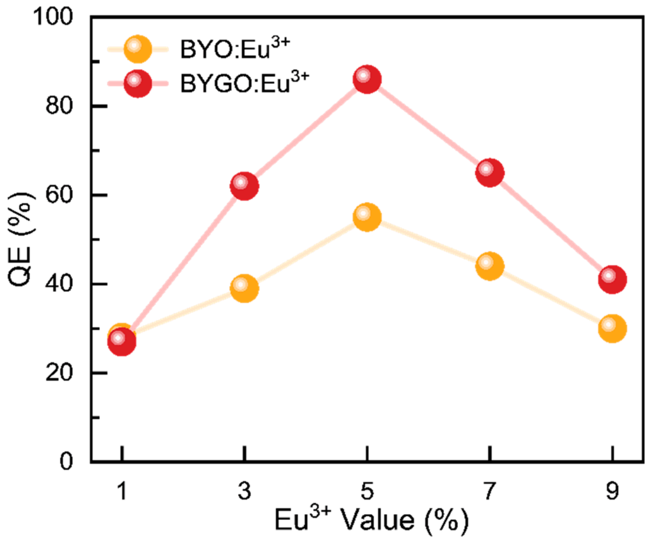

Figure S3 Comparison of QE between BYO: Eu^{3+} and BYGO: Eu^{3+}.

Figure S3 shows the variation trends of external quantum efficiency (EQ) for BYO and BYGO with different Eu^{3+} concentrations, with results similar to those in **Figure S2**. The quantum efficiency (QE) is defined as the ratio of the number of photons emitted as fluorescence to the number of absorbed excitation photons. It reflects the ability of a fluorescent material to convert absorbed excitation energy into fluorescent radiation. Under certain conditions, higher fluorescence efficiency typically correlates with higher luminescence intensity. When the Eu^{3+} concentration exceeds the quenching concentration of 5%, the fluorescence quantum efficiency decreases primarily due to enhanced non-radiative transitions caused by energy transfer and aggregation effects among the activator ions.

Figure S4

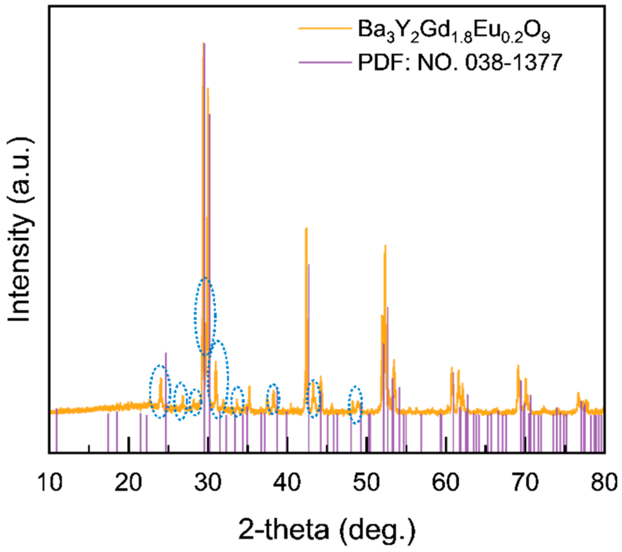

Figure S4 XRD of $Ba_3Y_2Gd_{1.8}Eu_{0.2}O_9$

Figure S4 shows the XRD pattern of the $Ba_3Y_2Gd_{1.8}Eu_{0.2}O_9$ phosphor, which is co-doped with 45% Gd^{3+} and 5% Eu^{3+}. The presence of excess Gd^{3+} that did not fully incorporate into the BYO lattice is indicated by the appearance of impurity phases, marked with a blue dashed line in the figure. As a result, the optimal doping concentration of Gd^{3+} was determined to be 40%, below which no phase transformation occurred

Table S1 The crystal structure data of $Ba_3Y_4O_9$, $Ba_3Y_{3.8}Eu_{0.2}O_9$ and $Ba_3Y_{2.2}Gd_{1.6}Eu_{0.2}O_9$ phosphors were analyzed based on the structural data of $Ba_3Y_4O_9$ provided by Szymanik et al. [1] in the PDF (012-0214) standard card as the reference theoretical values.

Space group	*R3 (146)*			
Lattice System	*Rhombohedral*			
Chemical Formula	$Ba_3Y_4O_9$	$Ba_3Y_{3.8}Eu_{0.2}O_9$	$Ba_3Y_{2.2}Gd_{1.6}Eu_{0.2}O_9$	Theory
Structural Density (g/cm^3)	5.585	5.757	5.963	5.520
a (Å)	6.0772604	6.1083171	6.1713445	6.110
b (Å)	6.0772604	6.1083171	6.1713445	6.110
c (Å)	25.2375641	25.4762192	25.6813860	25.460
α (◦)	90	90	90	90
β (◦)	90	90	90	90
γ (◦)	120	120	120	120
V(Å^3)	820.57962	824..45101	843.06898	823.10
Rwp	5.18	8.44	9.89	

Table S2 Diverse atomic parameters together with the refined atomic positions of $Ba_3Y_4O_9$ phosphor.

	Atomic Coordinate						
	x		y		z		Occ.
Site	Theory	This Work	Theory	This Work	Theory	This Work	
Ba_I	0	0	0	0	0.99182	0.91029	100%
Ba_{II}	0	0	0	0	0.41653	0.43814	100%
Ba_{III}	0	0	0	0	0.83885	0.87031	100%
Y_I	0	0	0	0	0.13445	0.14484	100%
Y_{II}	0	0	0	0	0.27235	0.29869	100%
Y_{III}	0	0	0	0	0.56833	0.56911	100%
Y_{IV}	0	0	0	0	0.70491	0.72537	100%
O_I	0.14934	0.13726	0.58169	0.58643	0.84638	0.86270	100%
O_{II}	0.10104	0.11159	0.06755	0.13960	0.75279	0.74486	100%
O_{III}	0.15668	0.14952	0.74943	0.70438	0.31470	0.30280	100%

Table S3 Diverse atomic parameters together with the refined atomic positions of $Ba_3Y_{3.8}Eu_{0.2}O_9$ phosphor.

	Atomic Coordinate						
	x		y		z		Occ.
Site	Theory	This Work	Theory	This Work	Theory	This Work	
Ba_I	0	0	0	0	0.99182	0.91029	100%
Ba_{II}	0	0	0	0	0.41653	0.43814	100%
Ba_{III}	0	0	0	0	0.83885	0.87031	100%
Y_I	0	0	0	0	0.13445	0.14484	91.30%
Eu_I							8.70%
Y_{II}	0	0	0	0	0.27235	0.29869	98.98%
Eu_{II}							1.02%
Y_{III}	0	0	0	0	0.56833	0.56911	98.99%
Eu_{III}							1.01%
Y_{IV}	0	0	0	0	0.70491	0.72537	91.22%
Eu_{IV}							8.78%
O_I	0.14934	0.19343	0.58169	0.61306	0.84638	0.85198	100%
O_{II}	0.10104	0.11398	0.06755	0.10823	0.75279	0.71943	100%
O_{III}	0.15668	0.13871	0.74943	0.70548	0.31470	0.30280	100%

Table S4 Diverse atomic parameters together with the refined atomic positions of $Ba_3Y_{2.2}Gd_{1.6}Eu_{0.2}O_9$ phosphor.

	Atomic Coordinate						
	x		y		z		Occ.
Site	Theory	This Work	Theory	This Work	Theory	This Work	
Ba_I	0.09703	0	0	0	0.99236	0.98178	100%
Ba_{II}	0	0	0	0	0.41653	0.41653	100%
Ba_{III}	0	0	0	0	0.83885	0.85328	100%
Y_I	0	0	0	0	0.13445	0.13463	34.31%
Gd_I							56.99%
Eu_I							8.70%
Y_{II}	0	0	0	0	0.27235	0.28566	83.35%
Gd_I							15.63%
Eu_{II}							1.02%
Y_{III}	0	0	0	0	0.56833	0.55577	81.42%
Gd_I							17.57%
Eu_{III}							1.01%
Y_{IV}	0	0	0	0	0.70491	0.71397	28.82%%
Gd_I							62.40%
Eu_{IV}							8.78%
O_I	0.14934	0.25636	0.58169	0.73103	0.84638	0.86166	100%
O_{II}	0.10104	0.06224	0.06755	0.18360	0.75279	0.68593	100%
O_{III}	0.15668	0.10507	0.74943	0.68907	0.31470	0.30026	100%

Table S5 Comparison of activation energy of the BYGO phosphor with some previously reported phosphors.

**Materials**	**E_a (eV)**	**References**
$NaBiF_4$:40%Eu^{3+}	0.237	[2]
$Gd_{3.67}Si_3O_{13}$:3%Eu^{3+}	0.28912	[3]
$NaGdMgTeO_6$:8%Eu^{3+}	0.2	[4]
$Gd_{0.9}InO_3$:10%Eu^{3+}	0.25	[5]
Ba_2GdTaO_6:10%Eu^{3+}	0.230	[6]
BYO:5%Eu^{3+}	0.1688	This work
BYGO:5%Eu^{3+}	0.3501	This work

Table S6 Colour temperature statistics of the samples

Temperature	CIE		Colour Temperature(K)	
	BYO:5%Eu^{3+}	BYGO:5%Eu^{3+}	BYO:5%Eu^{3+}	BYGO:5%Eu^{3+}
**300 K**	0.6382, 0.3258	0.6528, 0.3467	3161	2686
**325 K**	0.6346, 0.3240	0.6534, 0.3461	3168	2711
**350 K**	0.6303, 0.3212	0.6539, 0.3456	3203	2733
**375 K**	0.6052, 0.3051	0.6452, 0.3453	3438	2746
**400 K**	0.5996, 0.3021	0.6544, 0.3452	3468	2752
**425 K**	0.5937, 0.3007	0.6593, 0.3457	3404	2730
**450 K**	0.5951, 0.3008	0.6524, 0.3471	3432	2699

Note.S1 Quantum yields

Quantum efficiency (QE) is a crucial metric for assessing the quality of phosphors. It is defined as the ratio of the number of photons emitted by the phosphor to the number of photons absorbed. This ratio directly reflects the phosphor's ability to convert absorbed excitation energy into fluorescent radiation. A higher quantum efficiency indicates a stronger capability of the phosphor to transform excitation energy into photon emission, can be calculated by the following expression[7, 8]

$$QE = \frac{\int L_s}{\int E_r - \int E_s}$$

where L_s represents the emission spectrum of the phosphor, E_r represents the spectrum of the excitation light from the xenon lamp, and E_s represents the spectrum of the excitation light reflected from the sample.

Note S2 Decay time

Fluorescence decay time, also known as fluorescence lifetime, refers to the average time a fluorescent molecule spends in the excited state. It is the time required for the fluorescence intensity to decay from its maximum value to 1/e (approximately 36.8%) of its initial value. The fluorescence lifetime is an intrinsic property of the fluorescent molecule, reflecting the rate at which energy is lost from the excited state. This decay typically follows an exponential law, which can be mathematically expressed as[9, 10]:

$$I = A \times e^{\left(\frac{-t}{\tau_R}\right)} + B$$

Where τ_R is the fluorescence lifetime, t is the decay time, I is the relative fluorescence intensity, A and B are constants.

Note S3 Activation Energy

The activation energy for thermal quenching refers to the energy required for an excited-state electron to return to the ground state through non-radiative transitions. In the process of thermal quenching, the Arrhenius equation can be used to describe the relationship between the intensity of fluorescence or phosphorescence and temperature. Thermal quenching is a phenomenon where the efficiency of light emission decreases due to the loss of energy from the excited-state luminescent centers through non-radiative transitions (such as thermal relaxation) as the temperature increases. The calculation equation for thermal quenching is given by [2, 11]

$$I_T = I_0 \times e^{-\frac{E_a}{kT}}$$

where, k is the Boltzmann's constant (8.617105×10^{-5} eV/K), I_T and I_0 stand for the luminous intensity at target temperature and room temperature separately. This equation shows how the luminescence efficiency decreases with increasing temperature due to thermal quenching. The higher the activation energy E_a, the more stable the luminescence efficiency is against temperature increases, as it represents a higher energy barrier for non-radiative transitions.

Note S4 Colour temperature

Colour temperature (CCT) provides an accurate method for describing and distinguishing the colour of light emitted by phosphors. It is commonly used to assess the consistency of light colour from different phosphors or from the same phosphor under different conditions. The calculation of CCT is typically based on the CIE (International Commission on Illumination) standard chromaticity system. The CCT of each sample is determined according to the equation[8, 12, 13]

$$n = \frac{x - 0.332}{y - 0.1858}$$

$$T = -437n^3 + 3601n^2 - 6861n + 5514.31$$

where the x and y values represent the CIE coordinates and are detailed in **Table S6**.

Reference:

1. Szymanik, B; Buckley, R. G.; Trodahl, H. J. and Davis, R. L., Structure and decomposition of ceramic $Ba_3Y_4O_9$. *Solid State Ionics*, 1998, *109*, 223–228.

2. Du, P.; Huang, X.; Yu, J. S., Facile synthesis of bifunctional Eu^{3+}-activated $NaBiF_4$ red-emitting nanoparticles for simultaneous white light-emitting diodes and field emission displays. *Chem. Eng. J.* 2018, *337*, 91-100.

3. Ye, W.; Zhao, C.; Shen, X.; Ma, C.; Deng, Z.; Li, Y.; Wang, Y.; Zuo, C.; Wen, Z.; Li, Y.; Yuan, X.; Wang, C.; Cao, Y., High quantum yield $Gd_{4.67}Si_3O_{13}$: Eu^{3+} red-emitting phosphor for tunable white light-emitting devices driven by UV or blue LED. *ACS Appl. Electron. Mater.* 2021, *3*, 1403-1412.

4. Sreelekshmi, A. K.; Lal, S. C.; Ganesanpotti, S., Probing the multifunctionality of double layered perovskite $NaGdMgTeO_6$: Eu^{3+} in ratiometric phosphor thermometry and solid-state lighting. *J. Alloy. Compd.* 2022, *905*, 164138.

5. Wang, X.; Lu, B.; Xia, H., Novel red-emitting orthorhombic $GdInO_3$: Eu^{3+}perovskite phosphor: Structural resolution, Judd-Ofelt theory, and comparative investigation with hexagonal counterpart. *Materials Today Nano*, 2023, *21*, 100291.

6. Li, J.; Wang, X.; Cui, R.; Deng, C., Synthesis and photoluminescence studies of novel double-perovskite phosphors, Ba_2GdTaO_6: Eu^{3+} for WLEDs. *Optik*, 2020, *201*, 163536.

7. Qiao, J.; Zhang, S.; Zhou, X.; Chen, W.; Gautier, R.; Xia, Z., Near-infrared light-emitting diodes utilizing a europium-activated calcium oxide phosphor with external quantum efficiency of up to 54.7%. *Adv. Mater.* 2022, *34*, 2201887.

8. Wang, S.; Xu, Y.; Chen, T.; Jiang, W.; Liu, J.; Zhang, X.; Jiang, W.; Wang, L., A red phosphor $LaSc_3(BO_3)4$: Eu^{3+} with zero-thermal-quenching and high quantum efficiency for LEDs. *Chem. Eng. J.* 2021, *404*, 125912.

9. Yadav, R.; Khan, A. F.; Yadav, A.; Chander, H.; Haranath, D.; Gupta, B. K.; Shanker, V.; Chawla, S., Intense red-emitting $Y_4A_{l2}O_9$: Eu^{3+} phosphor with short decay time and high color purity for advanced plasma display panel. *Opt. Express*, 2009, *17*, 22023-22030.

10. Yuan, Y.; Yan, G.; Dreessen, C.; Rudolph, T.; Huelsbeck, M.; Klingebiel, B.; Ye, J.; Rau, U.; Kirchartz, T., Shallow defects and variable photoluminescence decay times up

to 280 μs in triple-cation perovskites. *Nat. Mater.* 2024, *23*, 391–397.

11. Guo, H.; Huang, X.; Zeng, Y., Synthesis and photoluminescence properties of novel highly thermal-stable red-emitting $Na_3Sc_2(PO_4)_3$: Eu^{3+} phosphors for UV-excited white-light-emitting diodes. *J. Alloy. Compd.* 2018, 741, 300-306.

12. Dang, P.; Li, G.; Yun, X.; Zhang, Q.; Liu, D.; Lian, H.; Shang, M.; Lin, J., Thermally stable and highly efficient red-emitting Eu^{3+}-doped $Cs_3GdGe_3O_9$ phosphors for WLEDs: non-concentration quenching and negative thermal expansion. *Light: Science & Applications* 2021, *10*, 29.

13. Wei, Y.; Yang, H.; Gao, Z.; Liu, Y.; Xing, G.; Dang, P.; Al Kheraif, A. A.; Li, G.; Lin, J.; Liu, R.-S., Strategies for designing antithermal-quenching red phosphors. Adv. Sci. 2020, *7*, 1903060.
